# CtBP modulates Snail-mediated tumor invasion in *Drosophila*

**DOI:** 10.1038/s41420-021-00516-x

**Published:** 2021-08-04

**Authors:** Chenxi Wu, Xiang Ding, Zhuojie Li, Yuanyuan Huang, Qian Xu, Rui Zou, Mingyang Zhao, Hong Chang, Chunhua Jiang, Xiaojin La, Gufa Lin, Wenzhe Li, Lei Xue

**Affiliations:** 1 The First Rehabilitation Hospital of Shanghai, Shanghai Key Laboratory of Signaling and Diseases Research, School of Life Science and Technology, Tongji University, 1239 Siping Road, Shanghai, 200092 China; 2 College of Traditional Chinese Medicine, North China University of Science and Technology, 21 Bohai Road, Tangshan, 063210 China; 3 College of Integrative Medicine, Fujian University of Traditional Chinese Medicine, Fuzhou, 350122 China; 4 Key Laboratory of Spine and Spinal Cord Injury Repair and Regeneration of Ministry of Education, Orthopaedic Department of Tongji Hospital, School of Life Sciences and Technology, Tongji University, Shanghai, 200092 China; 5 Zhuhai Interventional Medical Center, Zhuhai Precision Medical Center, Zhuhai People’s Hospital, Zhuhai Hospital Affiliated with Jinan University, Zhuhai, Guangdong 51900 China

**Keywords:** Cancer genetics, Cell migration

## Abstract

Cancer is one of the most fatal diseases that threaten human health, whereas more than 90% mortality of cancer patients is caused by tumor metastasis, rather than the growth of primary tumors. Thus, how to effectively control or even reverse the migration of tumor cells is of great significance for cancer therapy. CtBP, a transcriptional cofactor displaying high expression in a variety of human cancers, has become one of the main targets for cancer prediction, diagnosis, and treatment. The roles of CtBP in promoting tumorigenesis have been well studied in vitro, mostly based on gain-of-function, while its physiological functions in tumor invasion and the underlying mechanism remain largely elusive. Snail (Sna) is a well-known transcription factor involved in epithelial-to-mesenchymal transition (EMT) and tumor invasion, yet the mechanism that regulates Sna activity has not been fully understood. Using *Drosophila* as a model organism, we found that depletion of *CtBP* or *snail* (*sna*) suppressed Ras^V12^/*lgl*^*-/-*^-triggered tumor growth and invasion, and disrupted cell polarity-induced invasive cell migration. In addition, loss of *CtBP* inhibits Ras^V12^/Sna-induced tumor invasion and Sna-mediated invasive cell migration. Furthermore, both CtBP and Sna are physiologically required for developmental cell migration during thorax closure. Finally, Sna activates the JNK signaling and promotes JNK-dependent cell invasion. Given that CtBP physically interacts with Sna, our data suggest that CtBP and Sna may form a transcriptional complex that regulates JNK-dependent tumor invasion and cell migration in vivo.

## Introduction

Tumor metastasis is a major contributor to the high mortality rate of cancer and accounts for more than 90% of cancer-related fatalities in patients with palpable clinical traits^1,2^. Metastasis is a process of cancer cells disseminating from a primary lesion via lymphatic and/or blood circulations to distal organs, which involves a variety of cellular mechanisms^3^. These include invading through basement membranes (BM), escaping immune surveillance, modulating tissue microenvironment, and evolving resistance to therapeutic intervention^4–6^. Therefore, how to effectively control and reverse tumor invasion is of great significance to the clinical treatment of malignant cancer. Over the past decades, great progress has been made in exploring the mechanisms of tumor progression, in which numerous oncogenes, tumor suppressor genes, and multiple signaling pathways (e.g., Raf-MAPK, JNK, WNT, Hippo, Notch, JAK-STAT, and PI3K/AKT) have been implicated in tumor growth and invasion^7–10^. Among them, the oncogenic carboxy-terminal binding protein (CtBP) family members are the widely concerned because of their overexpression across an extensive spectrum of solid human tumors, including bladder, breast, ovarian, gastric, prostate, and sarcoma cancer, which have become the main targets for cancer prediction, diagnosis, and treatment^11–13^.

CtBP is a well-known and evolutionarily conserved transcriptional coregulator that was initially identified through its interaction with the human adenovirus E1A protein and plays a crucial role in regulating cell survival^14,15^. Although the invertebrate (nematodes, fruit flies, etc.) genome encodes a single CtBP protein, the vertebrate (mouse, rats, human, etc.) genome expresses two CtBP proteins (CtBP1 and CtBP2) that perform both unique and redundant functions^11^. Usually, CtBP binds to a variety of transcription repressors, such as Snail, Knirp, and Krüppel, through its N-terminal dehydrogenase PxDLS (Pro-x-Asp–Leu–Ser) motif, and recruits chromatin-modifying enzymes to form transcription repressor complex, which targets specific DNA promoter regions^16,17^. Extensive genetic and biochemical studies in model organisms have demonstrated that CtBP is indispensable for embryonic development and adult lifespan regulation. The homozygous mutation of *mCtBP2* in mouse leads to developmental defects and embryonic death, while *mCtBP1* homozygous deletion reduces their offsprings’ life span^18^. Conversely, loss of *CtBP* either by depletion or mutation triggered an extended life span in *C. elegans*^19^. In addition to its role in development, CtBP-mediated transcriptional coregulation plays important roles in a variety of diseases, especially cancer^20–22^. CtBP1/2 are highly expressed in several human cancer types, with their expression level correlating to the poor prognostic outcomes and aggressive tumor characteristics. In 2013, a comprehensive description of CtBP inhibition targets was achieved by the genome-wide analysis, in which the targets are mainly categorized into the following: genes that regulate DNA damage repair and genome stability, genes that regulate cell apoptosis and proliferation, and genes that regulate epithelial differentiation and impede epithelial-to-mesenchymal transition (EMT)^23^. Of note, signaling pathways related to these three categories' genes are generally dysregulated in cancer^20^. Besides, CtBP also functions as a coactivator to accelerate tumorigenesis by promoting cancer stem cell self-renewal^11^. However, despite many advances being made in elucidating the tumor-promoting effects of CtBP, most of the studies are based on gain of function, in vitro, or cell culture experiments, it remains poorly understood whether it could be the case in vivo. Whether endogenous CtBP is involved in cell invasion and EMT, or interacts with tumor-related signal pathways, as well as the potential molecular mechanisms, needs to be further explored.

Snail (Sna) belongs to the Sna transcriptional factor family, which was first identified in *Drosophila* as a critical regulator of mesoderm formation during embryonic development^24^. Snail family members have a conserved C-terminal DNA-binding domain containing four–six C_2_H_2_-type zinc fingers and a SNAG domain in N-terminus^25^. Generally, Sna acts as a transcriptional repressor through its SNAG domain to suppress the target gene expression^26^. Sna could also positively regulate transcription, and this functional switch of Sna may depend on its cofactor^27,28^. Sna has been extensively studied for its role in various biological processes, including embryonic development, cell fate decision, and cell differentiation^24,29–31^. In *Drosophila, sna* homozygous mutant embryos show defective in mesoderm formation^24^. Murine SNAI/SLUG interact with YAP/TAZ to control skeletal stem cell differentiation^32^. Besides, Sna is a well-known modulator of epithelial–mesenchymal transition (EMT) and tumor invasion^33^. SNAI is highly expressed in multiple types of cancer cell lines, and its expression correlates with increased metastasis potential. Sna family proteins regulate the transcription of a large number of genes essential for EMT and tumor progression^34,35^, for instance, SNAI directly represses the expression of epithelial markers like E-cadherin^36^, while upregulates that of mesenchymal markers like MMP2/9 to promote EMT^37^. Despite its well-established role in EMT and tumor invasion, the mechanism that regulates Sna activity in cancer progression has not been completely understood.

In this work, we found that *Drosophila* CtBP and Sna are physiologically required for Ras^V12^/*lgl*^*-/-*^ triggered tumor growth and invasion, and loss-of-cell polarity-induced invasive cell migration. We further showed that CtBP is indispensable for Sna-induced cell migration and tumor invasion. Moreover, Sna and CtBP regulate cell migration in thorax development. Finally, Sna activates the JNK signaling and promotes JNK-dependent cell migration. Taken together, these findings provide the in vivo evidences and the underlying mechanism for the role of CtBP in Sna-mediated cell migration and tumor invasion, and offer therapeutic strategies for clinical treatment of cancer and other related diseases.

## Results and discussion

### Loss-of-*CtBP* suppresses Ras^V12^/*lgl*^*-/-*^ induced tumor growth and invasion

**Fig. 1:**
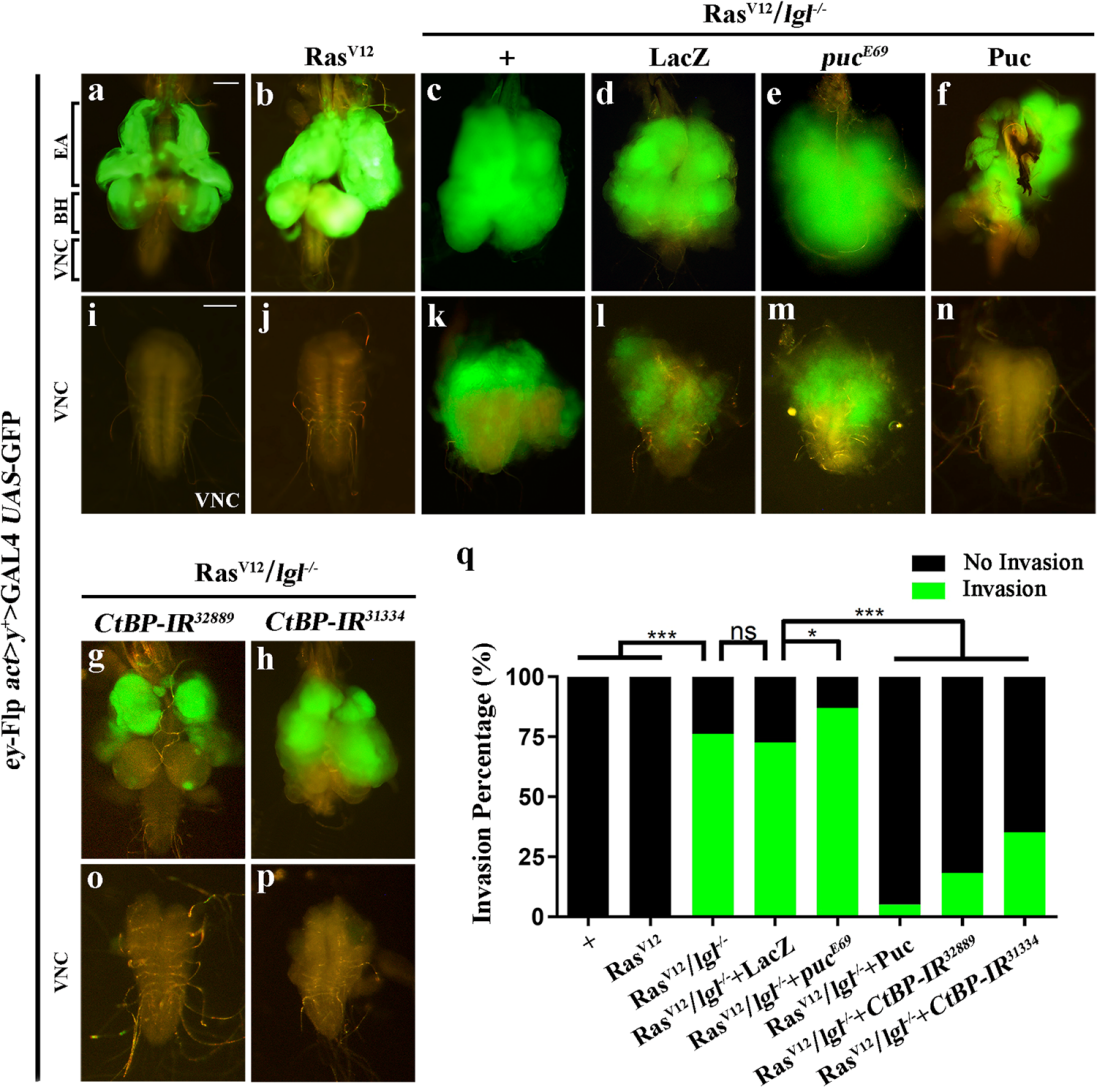
*CtBP* is necessary for Ras^V12^/*lgl*^*-/-*^-induced tumor growth and invasion. Fluorescent images showing *Drosophila* larval cephalic complexes (CC, **a**–**h**) and ventral nerve cords (VNC, **i**-p), the anterior is to the up in all panels. The CC (**a**) contains eye–antennal disks (EA), brain hemispheres (BH), and VNC (**h**). GFP-labeled mosaic clones were created in the EA. Compared with the control (**a**, **h**), Ras^V12^/*lgl*^*-/-*^ induced tumor overgrowth (**c**) and invasion to the VNC (**j**) were enhanced by *puc*^*E69*^ heterozygosity (**e**, **m**), and strongly suppressed by expressing Puc (**f**, **n**), or depleting *CtBP* (**g**, **o**, **h** and **p**), but not expressing LacZ (**d**, **l**). Ras^V12^-overexpressing clones showed a visible growth advantage (**b**), but did not migrate to the VNC (**j**). Statistical analysis of the invasion percentage (**q**) as shown in figures **a**–**h** (**a**, 0.00%, *n* = 52; **b**, 0.00%, *n* = 55; **c**, 76.27%, *n* = 118; **d**, 72.58%, *n* = 124; **e**, 86.96%, *n* = 92; **f**, 5.09%, *n* = 118; **g**, 18.33%, *n* = 120; **h**, 35.19%, *n* = 108), respectively. Chi-squared test was applied to compute *P*-values, *P < 0.05, ***P < 0.001; ns, no significant difference. See the electronic supplementary material for detailed genotypes. Scale bar: 100 µm (**a**–**p**).

In line with previous studies^38,39^, clones of GFP-marked wild-type cells mediated by *eyeless* (*ey*)-Flp/MARCM system were observed in the larval eye–antennal imaginal disks and the brain optic lobes (Fig. 1a and Supplementary Fig. 1a), but were not seen in the adjacent ventral nerve cord (VNC) of the central nervous system (Fig. 1i). While the GFP-labeled clones expressing activated Ras (Ras^V12^) alone caused noticeable growth without invading to the VNC (Fig. 1b, j; Supplementary Fig. 1b), ectopic expression of Ras^V12^ in *lgl*^*4*^ homozygous mutant (*lgl*^*-/-*^) mosaic clones resulted in massive tumor-like overgrowth (Fig. 1c; Supplementary Fig. 1c) and invasive metastasis to the VNC (Fig. 1k, q). Besides, the invasive tumor cells triggered an extended larval stage, which impeded the normal development of larvae into pupae, and animals died as bloated third instar larvae (Supplementary Fig. 1c, h). These phenotypes, mediated by the c-Jun N-terminal kinase (JNK) pathway^40^, were modestly enhanced by *puc*^*E69*^ heterozygosity and effectively blocked by the expression of Puckered (Puc) (Fig. 1e, f, m and n; Supplementary Fig. 1e, h), a phosphatase and inhibitor of JNK^41^. Consistently, the expression of *puc-*LacZ, a reporter of JNK pathway, was strongly upregulated in Ras^V12^/*lgl*^*-/-*^ tumor cells, compared with control or Ras^V12^ clones (Supplementary Fig. 2c–e).

Using this Ras^V12^/*lgl*^*-/-*^ in vivo tumor model, we have conducted a genetic screen for regulators of tumor growth and invasion^39,42–45^. We found that the tumor overgrowth and metastasis phenotypes were strongly suppressed by RNA interference (RNAi)-mediated knockdown of *CtBP* (Fig. 1g, o, q; Supplementary Fig. 1f, h). To exclude the possibility of off-target effect from RNAi, we obtained from the Bloomington *Drosophila* stock center another independent *CtBP* RNAi line that targets a distinct region of the *CtBP* transcript^46^, and observed a similar, albeit moderate, suppression on tumor growth and invasion to VNC (Fig. 1h, p and q). Meanwhile, expression of *CtBP* RNAi in otherwise wild-type clones had no effect on the clonal growth (Supplementary Fig. 1g and Supplementary Fig. 3). qRT-PCR assay was performed to verify the knockdown efficiencies of the *CtBP* RNAi lines (Supplementary Fig. 2a). To exclude the possibility that the suppression effect of *CtBP* RNAi is a result of UAS titration, *UAS*-LacZ was included as a negative control (Fig. 1d, l and q and Supplementary Fig. 1d, h). Consistent with its role in Ras^V12^/*lgl*^*-/-*^-triggered tumor progression, we found that *CtBP* expression was increased in tumors (Supplementary Fig. 2b). Collectively, these results indicate that the transcriptional corepressor CtBP plays an essential role in Ras^V12^/*lgl*^*-/-*^ promoted tumor growth and invasion.

To examine whether loss-of-*CtBP* suppresses tumor invasion by increasing cell death or reducing cell proliferation, we checked cell death by cDcp-1 antibody staining and cell proliferation by Phospho-Histone H3 (PH3) staining in *CtBP* null mutant clones. Loss-of-*CtBP* did not cause enhanced cell death (Supplementary Fig. 2f–g) or reduced cell proliferation (Supplementary Fig. 4), suggesting that CtBP regulates tumor invasion independent of cell death and cell proliferation.

### CtBP is required for disrupted cell polarity-induced cell invasion

**Fig. 2:**
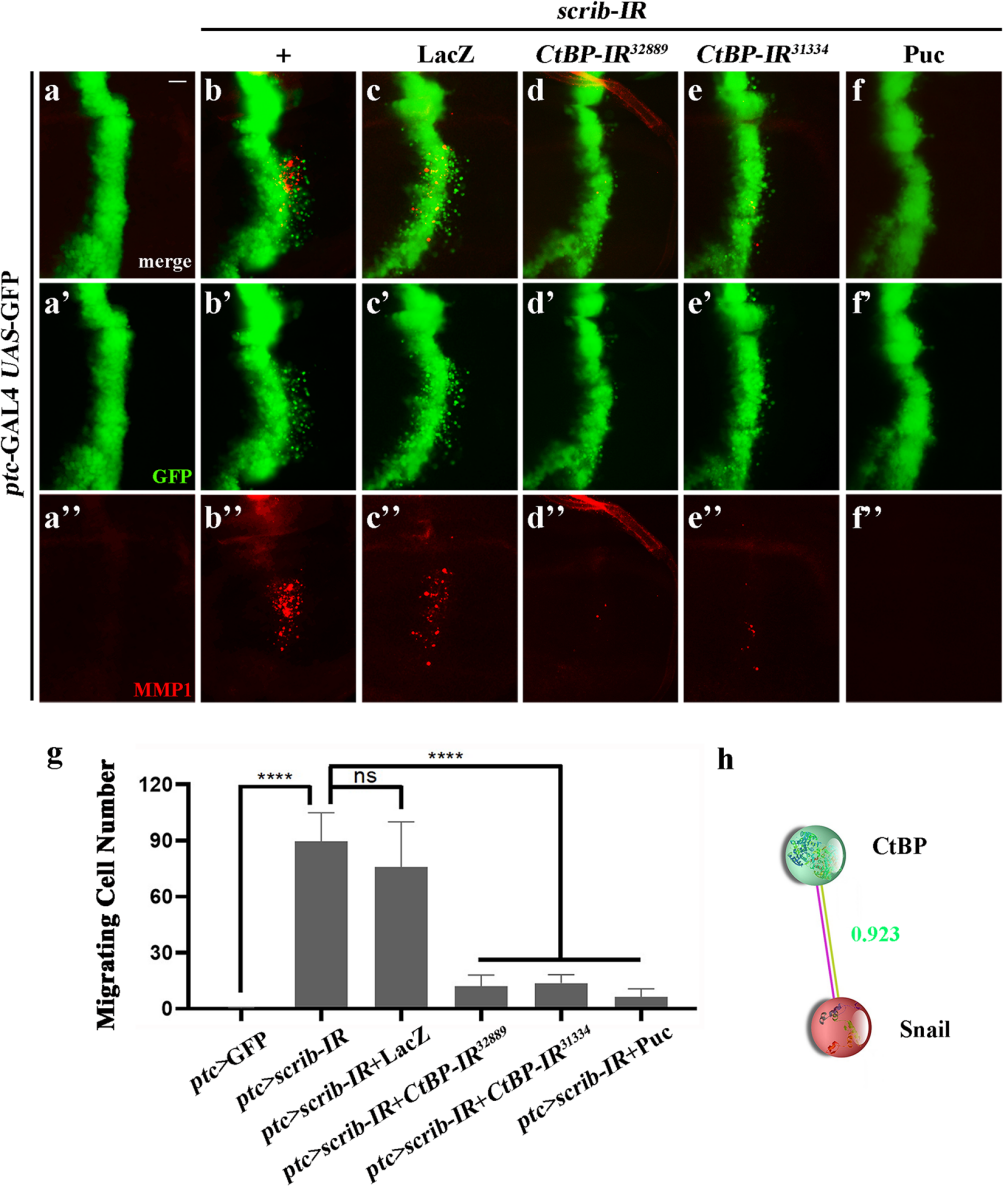
*CtBP* is required for cell polarity disruption-induced cell migration. **a**–**f** Fluorescence micrographs of 3^rd^ instar larval wing disks stained with anti-MMP1 antibody are shown, anterior is to the left and cells are marked with GFP expression. Compared with the control (**a**–**a”**), loss of *scrib* induced intensive cell migration and MMP1 upregulation (**b**–**b”**), which remained unchanged by expressing LacZ (**c**–**c”**), but was dramatically impeded by knockdown of *CtBP* (**d**–**d”**, **e**–**e”**), or expression of Puc (**f**–**f”**). **g** Column bar graph of the migrating cell number in **a**–**g** (*n* = 10 for each genotype; **a**, mean = 0.02; **b**, mean = 89.50; **c**, mean = 76.00; **d**, mean = 12.10; **e**, mean = 13.75; **f**, mean = 6.40), error bars indicate standard deviation. One-way ANOVA with Bonferroni multiple comparison test was used to compute *P*-values, ****P < 0.0001; ns, no significant difference. **h** Schematic diagram of the interaction between CtBP and Sna (generated by STRING 11.0 online analysis platform). Colored nodes: query proteins and first shell of interactors; filled nodes: 3D structure is known or predicted; rose red line: experimentally determined; dark-yellow line: textmining. The combined interaction score is 0.923. See the electronic supplementary material for detailed genotypes. Scale bar: 20 µm (**a**–**f**).

To verify the physiological function of *CtBP* in cell invasion, we employed another well-established invasion model^42,47^. In the epithelia of *Drosophila* larval wing imaginal disks, knockdown of cell polarity genes, e.g., *scrib*, *lgl*, or *dlg*, driven by *patched* (*ptc*)-GAL4 in the anterior/posterior (A/P) compartment boundary, induced a JNK-dependent cell invasion phenotype^43,48^. Consistently, GFP-marked depletion-of-*scrib* resulted in broadscale cell migration toward the P compartment, coupled with upregulation of matrix metalloprotease 1 (MMP1) (Fig. 2a–a”, b–b”), which is essential for basement membrane degradation and a molecular feature of EMT^49,50^. We found that *ptc* > *scrib-IR*-triggered cell invasion and MMP1 activation were notably blocked by depletion of *CtBP* (Fig. 2d–d”, e–e”) or expression of Puc (Fig. 2f–f”), but remained unaffected by LacZ expression (Fig. 2c–c”). To quantify this phenotype, we counted the total number of migrating cells in the wing pouch region, and found that depletion of *CtBP* reduced the number by 86.48% (*CtBP-IR*^*32889*^) or 84.64% (*CtBP-IR*^*31334*^), which is comparable to that of Puc expression (92.85%), while LacZ served as a negative control (Fig. 2g). Taking these data together, we conclude that CtBP is required for cell polarity disruption-triggered cell invasion and MMP1 upregulation.

### An evolutionary conserved role of Sna in tumor invasion

To uncover the mechanism by which CtBP modulates tumor invasion, we considered the transcription factor Snail (Sna) as a putative factor that cooperates with CtBP. First, Sna can interact with CtBP through its Pro-X-Asp–Leu–Ser-X-Lys (P-DLS-K) motif, and then recruit chromatin-modifying enzymes to form transcription repressors that bind to the promoter regions of target genes during development^51,52^. Second, the interaction score between Sna and CtBP, generated by the STRING 11.0 online analysis platform (http://string-db.org), is 0.923 with a high confidence (Fig. 2h). Third, previous studies reported that Sna is involved in controlling EMT during tumor progression, whose expression correlates with the tumor grade, nodal metastasis of multiple tumors, and indicates a poor outcome in patients with malignant tumor^53^.

**Fig. 3:**
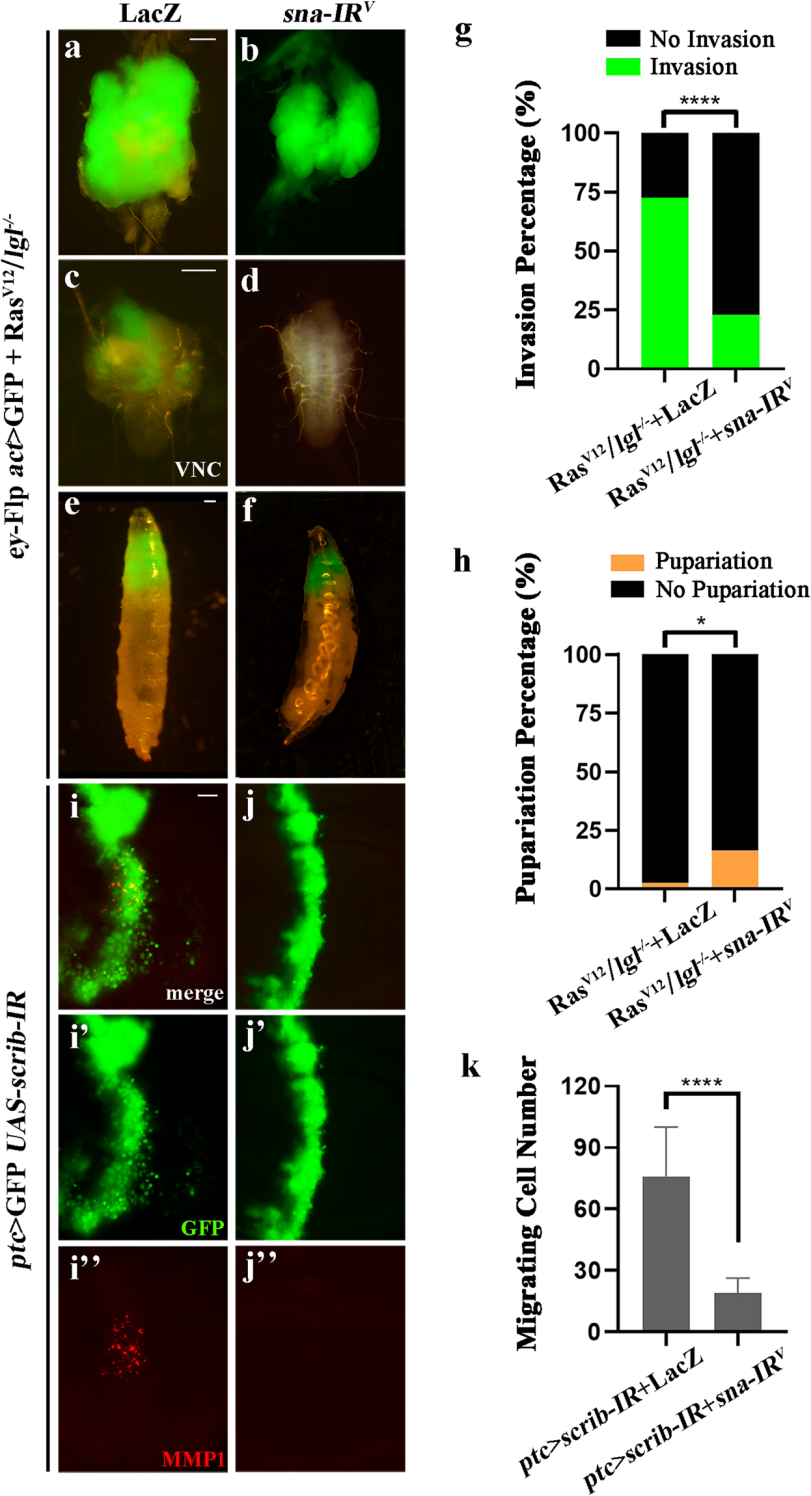
*sna* is required for tumor invasion and cell migration. (**a**–**f**, **i**–**i**” and **j**–**j**”) Fluorescence micrographs of larval CCs (**a**, **b**), VNCs (**c**, **d**), whole bodies (**e**, **f**) and wing discs (**i**–**i**”, **j**–**j**”) are shown. Compared with the LacZ controls (**a**, **c**, **e** and **i**–**i**’’), Ras^V12^/*lgl*^−^^/^^−^-induced tumor growth and invasion, and *ptc*>*scrib*-*IR*-triggered cell migration and MMP1 expression were notable blocked by expressing a *sna* RNAi (**b**, **d**, **f** and **j**–**j**’’). Statistical analysis of the invasion percentage (**g**), the pupariation percentage (**h**) and the migrating cell number (**k**) as shown in figures **a**–**b** (**a**, 72.58%, *n* = 124; **b**, 22.81%, *n* = 114), **e**–**f** (**e**, 2.56%, *n* = 39; **f**, 16.36%, **n** = 55) and **i**–**j** (*n* = 10 for each genotype; **i**, mean = 76.00; **j**, mean = 19.00) were shown. Chi-squared test or two tailed unpaired t-test was applied to compute *P*-values, *P < 0.05, ****P < 0.0001. See the electronic supplementary material for detailed genotypes. Scale bar: 100 µm (**a**–**f**), 20 µm (**i**–**j**).

**Fig. 4:**
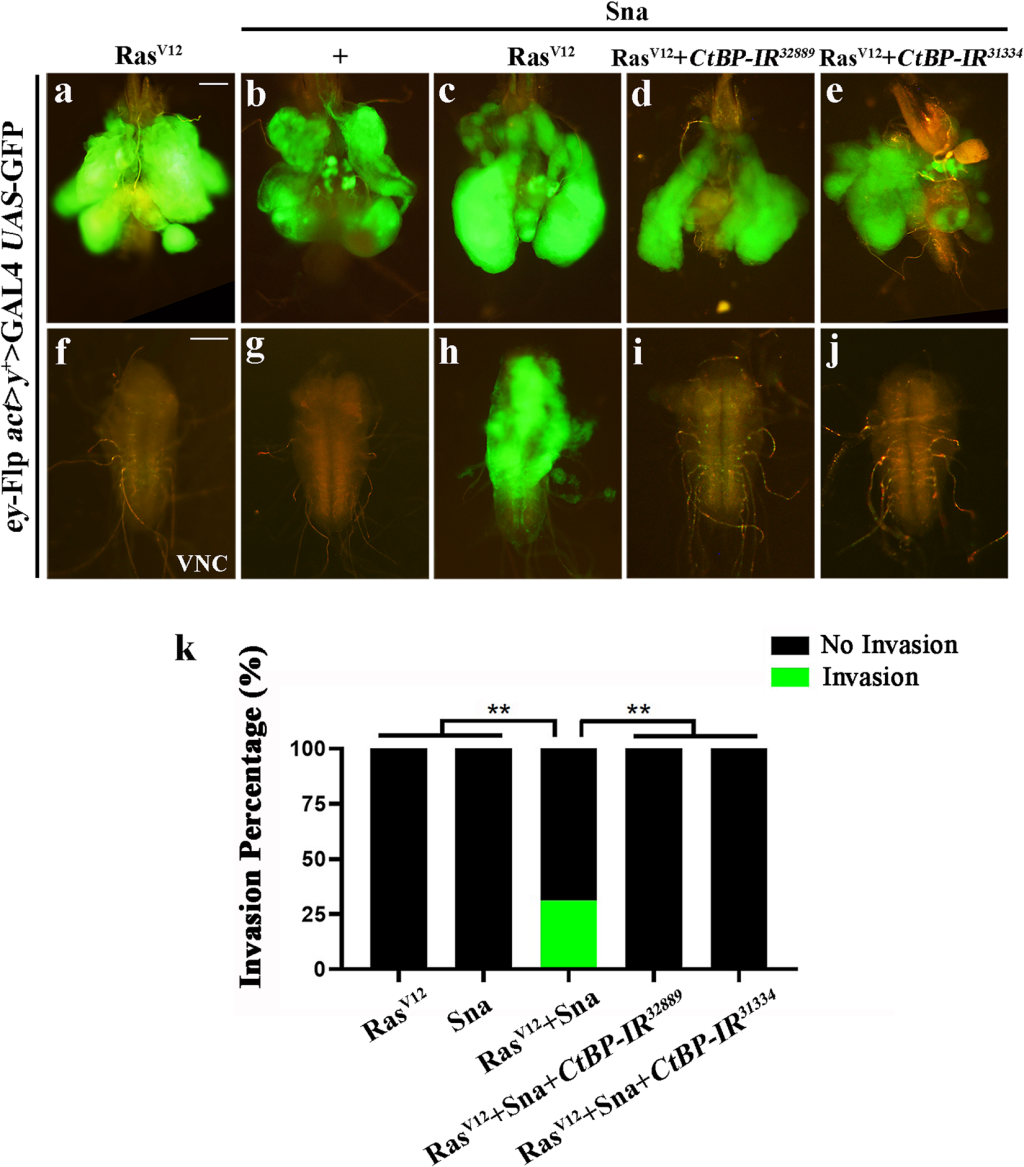
*CtBP* is essential for Ras^V12^/Sna-triggered tumor invasion. Fluorescent images of larval CCs (**a**–**e**) and VNCs (**f**–**j**). Ras^V12^ clones showed an obvious growth advantage (**a**, **f**), while overexpression of Sna alone did not promote any tumor-like phenotype (**b**, **g**). Coexpression of Sna and Ras^V12^ triggered moderate tumor overgrowth and VNC invasion (**c**, **h**), which was totally inhibited by knockdown of *CtBP* (**d**, **e**, **i** and **j**). **k** Column bar graph of the invasion percentage as shown in figures **a**–**e** (**a**, 0.00%, *n* = 55; **b**, 0.00%, *n* = 36; **c**, 31.11%, *n* = 45; **d**, 0.00%, *n* = 43; **e**, 0.00%, *n* = 27). Chi-squared test was applied to compute *P*-values, **P < 0.01. See the electronic supplementary material for detailed genotypes. Scale bar: 100 µm (**a**–**j**).

Although the role of Sna in tumor invasion has been well studied in mammals, it remains unknown whether this function is conserved in *Drosophila*. To test this, we first examined the physiological role of Sna in the Ras^V12^/*lgl*^*-/-*^ tumor model. Based on previous work^54^, we selected from Vienna *Drosophila* RNAi center (VDRC) a *sna* RNAi line with a high knockdown efficiency, and found that depletion of *sna* significantly inhibited Ras^V12^/*lgl*^*-/-*^ triggered tumor growth and invasion (Fig. 3a–d and g), and 16.36% larvae successfully developed into pupal stage (Fig. 3e, f and h). Furthermore, *ptc* > *scrib-IR*-induced cell invasion and MMP1 upregulation were significantly suppressed by depleting *sna* (Fig. 3i–i”, j–j” and k). Finally, we investigated the ability of Sna to promote tumor invasion in *Drosophila*. While ectopic expression of Sna alone in the eye disks failed to induce any tumor-like growth and invasion (Fig. 4b, g and k), it is sufficient to promote the invasion of Ras^V12^-expressing cells into the VNC (Fig. 4a, c, f, h and k). Thus, the critical role of Sna in tumor invasion has been conserved from fly to human.

### CtBP is essential for Sna-mediated tumor invasion and EMT

To dissect the role of CtBP in Sna-mediated tumor invasion, we employed the Ras^V12^/Sna tumor model, and found that Ras^V12^/Sna-triggered tumor invasion was remarkably impeded by *CtBP* knockdown (Fig. 4d, e and i–k), suggesting that CtBP is essential for Ras^V12^/Sna-triggered tumor invasion.

**Fig. 5:**
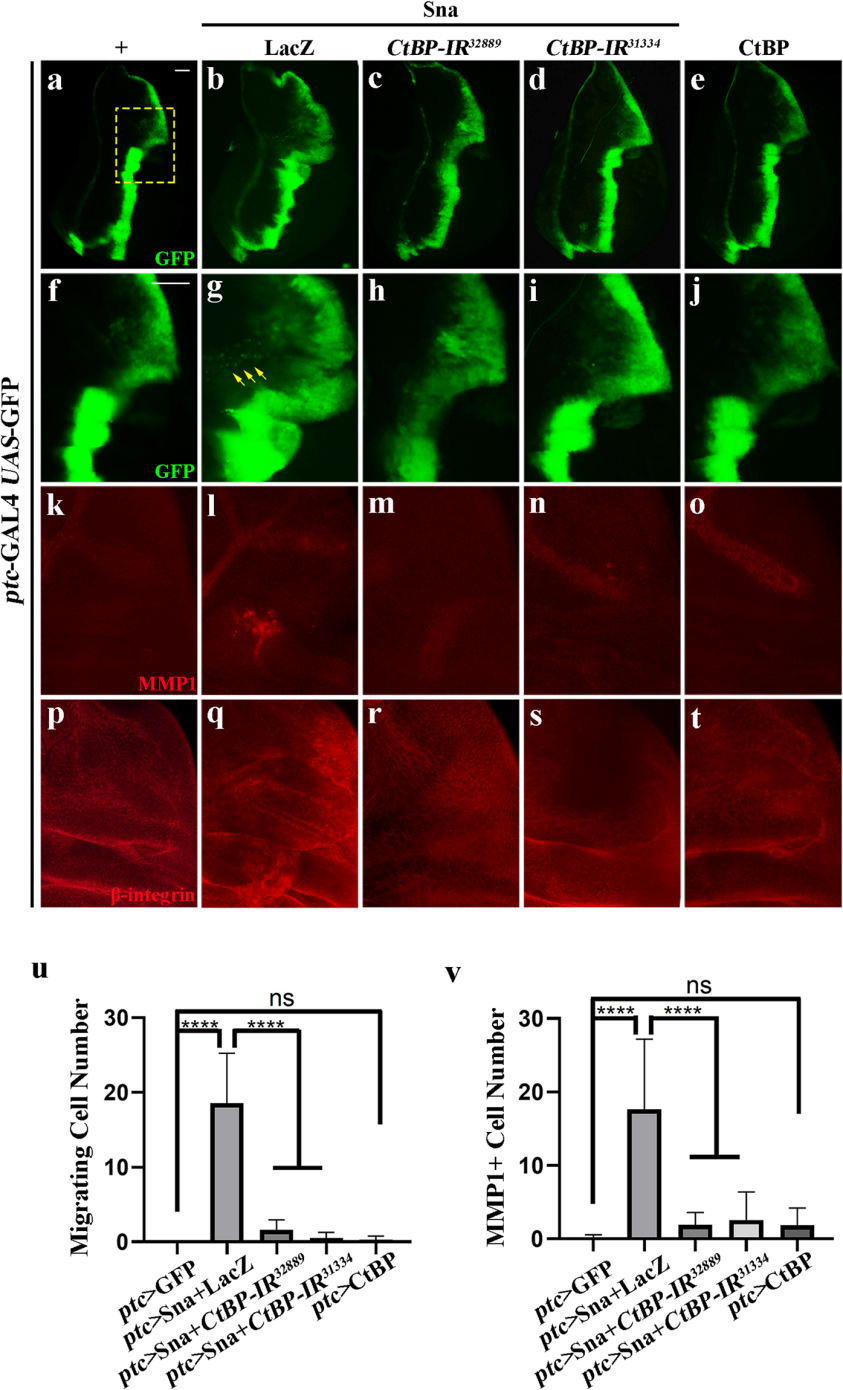
*CtBP* is indispensable for Sna-induced invasive cell migration. (**a**–**t**) Fluorescence micrographs of 3^rd^-instar larval wing disks are shown. Anterior is to the left and dorsal up. The individual channels detecting only GFP (green, **a**–**e** and **f**–**j**), only MMP1 (red, **k**–**o**), and only β-integrin signal (red, **p**–**t**). **f**–**j**, **k**–**o** and **p**–**t** are high magnification of the yellow-dotted boxed areas in **a**–**e**. *ptc*-GAL4 *UAS*-GFP is the control (**a**, **f**, **k** and **p**). Ectopic expression of Sna-induced cell migration (**g**, yellow arrows indicate the migrated cells), MMP1 upregulation (**l**), and β-integrin accumulation (**q**) was impeded by RNAi-mediated inactivation of *CtBP* (**h**, **i**, **m**, **n**, **r** and **s**). While expression of CtBP alone did not produce visible defects during larval stage (**e**, **j**, **o** and **t**). Statistical analysis of migrating cell number (**u**) and MMP1 + cell number (**v**), error bars indicate standard deviation. One-way ANOVA with Bonferroni multiple-comparison test was used to compute *P*-values, ****P < 0.0001; ns, no significant difference. See the electronic supplementary material for detailed genotypes. Scale bar: 40 µm (**a**–**t**).

Epithelial–mesenchymal transition (EMT) is a crucial step toward tumor metastasis, which endows cells with the capacity to break through basement membranes, resolve out cell–cell junctions, and migrate away from their initial site^55,56^. In mammals, Sna family members are regarded as the major transcription factors governing EMT^53^. To check whether this EMT-promoting function is conserved by *Drosophila* Sna, we overexpressed Sna along the A/P compartment boundary by *ptc*-GAL4. Consistently, we observed a conspicuous expansion of the GFP stripe, mostly notable in the dorsal region (Fig. 5b, u), with some GFP-positive cells migrating toward anterior (Fig. 5g), accompanied by EMT hallmarks, including MMP1 elevation (Fig. 5l, v) and β-integrin accumulation (Fig. 5q). These Sna-induced EMT features were significantly suppressed by knockdown of *CtBP* (Fig. 5c, d, h, i, m, n, r and s). However, ectopic expression of CtBP failed to produce any EMT-like phenotype (Fig. 5e, j, o and t). Thus, we conclude that CtBP is necessary, but not sufficient, for Sna-mediated EMT processes.

### Sna promotes cell invasion independent of cell death

Sna is also known to regulate cell death, while the role of cell death in Sna-induced cell invasion has not been explored. To this end, we performed cDcp-1 staining and found that *ptc* > *scrib-IR*-triggered cell death was partially suppressed by depletion of *sna* (Supplementary Fig. 5a–c). In addition, ectopic expression of Sna was sufficient to induce apoptosis, mostly in the dorsal region (Supplementary Fig. 5d), where invasion was observed (Fig. 2b and g). However, blocking apoptosis by overexpressing P35 did not affect Sna-induced cell invasion and MMP1 activation (Supplementary Fig. 6a–a”, c–c”). To further distinguish Sna-triggered cell invasion from apoptosis-induced proliferation (AiP), we expressed Dronc^DN^ to interfere the function of endogenous Dronc, which plays a key role in AiP. Blocking AiP had no effects on Sna-induced cell invasion and MMP1 upregulation (Supplementary Fig. 6b–b”). Collectively, these results suggest that Sna-triggered cell invasion is independent of cell death.

### Sna and CtBP regulate cell migration in normal development

**Fig. 6:**
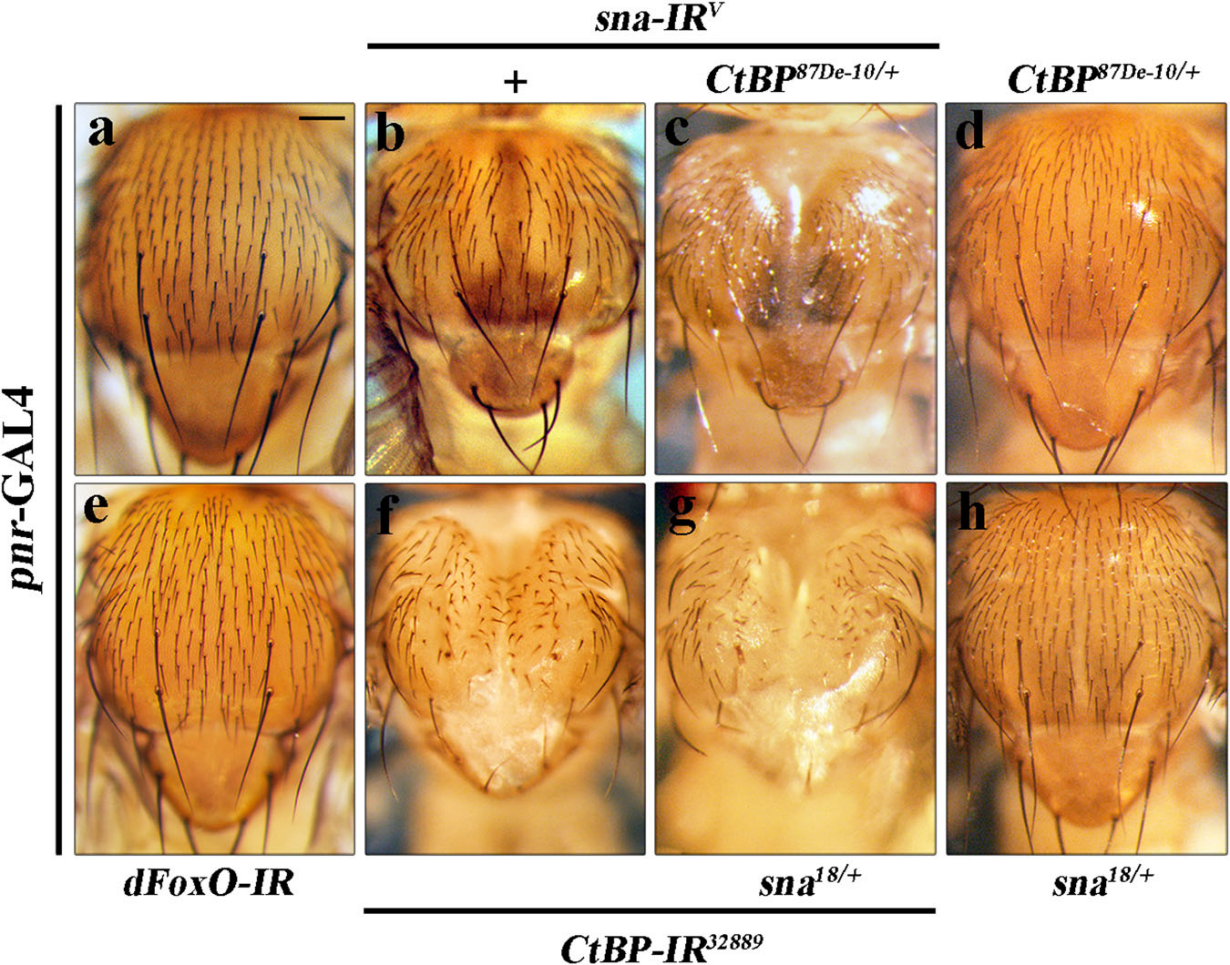
Sna and CtBP participate in thorax closure during development. Light micrographs of *Drosophila* adult thoraxes are shown. Compared with the control (**a**), depletion of *sna* (**b**) or *CtBP* (**f**) induced thorax cleft in development. Mutating one copy of endogenous *CtBP* (**d**) or *sna* (**h**) produced no obvious phenotype, but synergistically aggravated the *pnr* > *sna-IR* (**c**) or *pnr* > *CtBP-IR*- (**g**) triggered thorax closure defects, respectively. Knockdown of *dFoxO* showed no obvious defects, which was used as a negative control (**e**). See the electronic supplementary material for detailed genotypes. Scale bar: 100 µm (**a**–**h**).

In *Drosophila*, thorax closure is another remarkable model to study epithelial cell migration in development^57^. To investigate whether CtBP and Sna regulate cell migration in normal development, we knocked down either gene by the thorax-specific *pannier* (*pnr*)-GAL4 driver. Intriguingly, we found that *sna* depletion resulted in a mild cleft phenotype in the thorax (Fig. 6b), which was enhanced by heterozygosity for *CtBP* mutation (Fig. 6c). Likewise, RNAi-mediated downregulation of *CtBP* induced a thorax cleft phenotype (Fig. 6f), which was exacerbated in heterozygous *sna* mutants (Fig. 6g). On the other hand, heterozygosity for *sna* or *CtBP* (Fig. 6d, h), or knockdown of an unrelated gene *dFoxO* (Fig. 6e), gave no distinguishable phenotype. Collectively, these evidences suggest that Sna and CtBP may function together to regulate cell migration in thorax development.

### Sna promotes JNK pathway activation

**Fig. 7:**
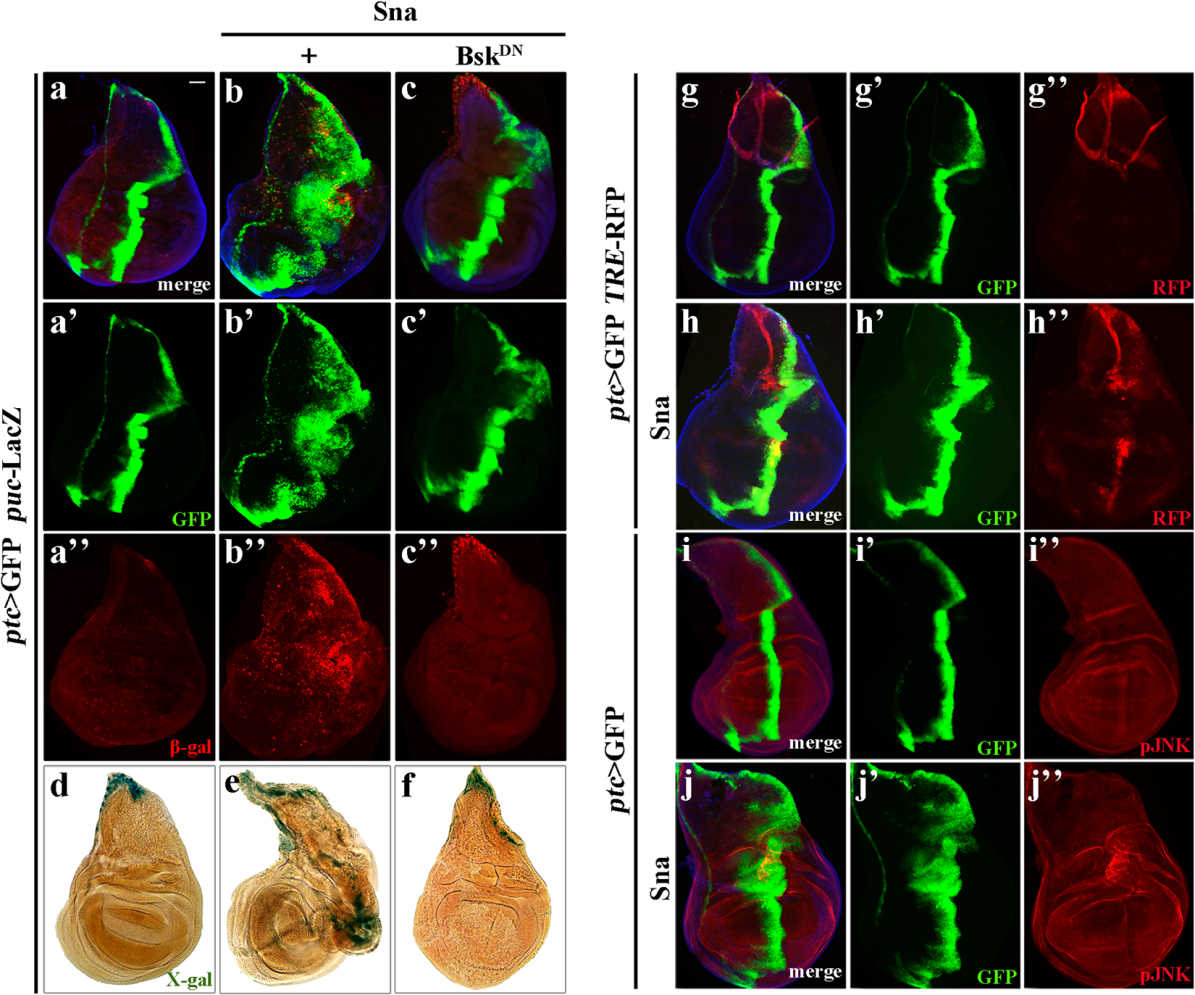
Sna activates JNK signaling in vivo. **a**–**c** and **g**–**j** Merged fluorescence micrographs of *Drosophila* third-instar larval wing disks are shown. The individual channels detecting only GFP (green, **a’**–**c’** and **g’**–j**’**), β-gal (red, **a”**–**c”**), RFP (**g”**, **h”**), or pJNK signal (red, **i”**–**j”**). (**d**–**f**) Light micrographs showing X-gal staining of the *puc*-LacZ reporter in wing disks. Compared with the controls (**a**, **d**, **g** and **i**), ectopic expression of Sna elevated the expression of *puc*-LacZ (**b**, **e**), *TRE*-RFP (**h**), and JNK phosphorylation (**j**). The increased *puc* transcription triggered by *ptc* > Sna is largely impeded by expressing Bsk^DN^ (**c**, **f**). See the electronic supplementary material for detailed genotype. Scale bar: 40 µm (**a**–**f**).

The JNK pathway plays crucial roles in regulating cell migration and tumor invasion^8,39^. To investigate the mechanism that underlies Sna-induced EMT, we checked the activity of JNK signaling. Compared with the *ptc* > GFP control (Fig. 7a–a”, d), ectopic expression of Sna dramatically enhanced the expression of a puc-LacZ reporter by executing an antibody-staining (Fig. 7b–b”) or X-gal staining assay (Fig. 7e), which was abolished by expressing a dominant negative form of the *Drosophila* JNK ortholog Bsk (Bsk^DN^, Fig. 7c–c”, f). *TRE*-RFP, which carries multiple binding sites for the AP-1 (Jun/Fos) transcription complex, is another reporter of JNK signaling^58^. Compared with the control (Fig. 7g–g”, i–i”), expressing Sna was sufficient to upregulate *TRE*-RFP expression (Fig. 7h–h”), and induce JNK phosphorylation detected by a specific anti-pJNK antibody (Fig. 7j–j”). Of note, the *puc*-LacZ reporter is a LacZ-bearing P-element inserted into the second intron of *puc*, and hence, acts as a loss-of-function allele (also known as *puc*^*E69*^). Intriguingly, *ptc* > Sna-induced cell migration (Fig. 7j’) was significantly enhanced by loss-of-*puc* (Fig. 7b’), but suppressed by Bsk^DN^, suggesting that Sna promotes JNK-dependent cell migration in *Drosophila*.

## Conclusions

Most cancer-related deaths are caused by secondary tumors formed through invasion, a rather complex and poorly understood process. With the multiple genetic tools and conserved tumor invasion machinery, *Drosophila* has been widely considered as an outstanding model organism to explore the invasion program^43,44,48^. The CtBP protein is a well-characterized and evolutionarily conserved transcriptional corepressor that plays crucial roles during development and oncogenesis. In this work, we identified CtBP as a novel regulator of Ras^V12^/*lgl*^*-/-*^ induced tumor growth and invasion. Besides, CtBP is also required for loss-of-cell polarity*-*triggered cell invasion in the wing disks, and developmental cell migration in thorax closure. Mechanistically, CtBP may interact with Sna to form a transcriptional complex that activates the JNK signaling and promotes JNK-dependent cell migration and tumor invasion. Yet, the contribution of CtBP and JNK in Sna-induced EMT needs to be verified in human cancers, which may provide additional drug targets and therapeutic strategies for clinical treatment of malignant tumors.

## Materials and methods

### Fly strains

Flies were kept on a cornmeal and agar medium at 25 °C according to standard protocols unless indicated. For producing the fluorescently labeled invasive tumors in the eye disks, the following strains were previously described^38,39,44^, including *yw ey*-Flp; *tub*-GAL80 FRT40A; *act* > *y*^*+*^>GAL4 *UAS*-GFP (40 A tester), *lgl*^*4*^ FRT40A *UAS*-Ras^V12^ (40 A tester), and *ey*-Flp *act* > *y*^*+*^>GAL4 *UAS*-GFP and *UAS*-Ras^V12^. Additional *Drosophila* strains used, including *UAS-CtBP-IR* (32889 and 31334), *sna*^*18*^ (3299), and *UAS-dFoxO-IR* (27656), were obtained from Bloomington *Drosophila* stock center. *UAS-sna-IR*^*V*^ (6263) and *UAS*-*scrib-IR* (27424) were received from Vienna *Drosophila* RNAi center (VDRC). *ptc*-GAL4^59^, *UAS*-Puc, *UAS*-Bsk^DN^, *puc*-LacZ^60^, *UAS*-LacZ, *UAS*-GFP, *UAS*-Sna^74b^ ^54^, *TRE*-RFP^58^, *pnr-*GAL4, *UAS*-Dronc^DN^, and *UAS*-P35 were previously described^42,43,47,61^. *UAS*-CtBP and *CtBP*^*87De-10*^ were kind gifts from Professor Ming Fang^62,63^. For all fly cross-experiments, healthy unmated male and female parents were randomly assigned to different groups. Double-blinded method was employed during the experiments.

*CtBP* mutant clones were generated in 3^rd^ instar larval eye disk by using the following strains: *ey*-Flp *act* > y^+^ > Gal4 *UAS*-GFP; *FRT82B tub-*Gal80 (82B MARCM tester) and *FRT82B CtBP*^*87De-10*^.

For *ptc* > GFP + *scrib-IR* cell migration experiments, animals were reared at 25 °C for 2 days, then shifted to 29 °C for additional 3 days, and the wing disks were dissected from 3^rd^-instar larvae^44^. For *ptc* > GFP + Sna migration assays, as ectopic expression of Sna is too strong to cause lethality before reaching the third-instar larva stage, animals were maintained at 18 °C.

### qRT-PCR

For RNAi-knockdown efficiency experiments, *hs*-Gal4 driver was used. Animals were raised at 25 °C, heat-shocked at 37 °C for 30 min, and recovered at 29 °C for 2 h before dissection.

Total RNAs were isolated from third-instar larval eye disk, and qRT-PCR was performed as previously described^64^. *rp49* served as an internal control.

Primers used are provided:

*rp49-*FP: TACAGGCCCAAGATCGTGAA.

*rp49-*RP: TCTCCTTGCGCTTCTTGGA.

*CtBP-*FP: GTCATCTTCTACGATCCCTACCT.

*CtBP-*RP: GCAATCGGACTGGAAAAGCA.

### Immunostaining

Dissected disks were fixed in 4% formaldehyde for 20 min. After several washes with 0.3% (v/v) PBST, disks were stained with primary antibodies at 4 °C overnight and then with secondary antibodies at room temperature for 2 h. The following antibodies were used: rabbit anti-cDcp-1 (1:100, Cell Signaling Technology, CST, Cat. #9578), rabbit anti-Phospho-Histone H3 (1:400, CST, Cat. #9701), mouse anti-MMP1 (1:200, Developmental Studies Hybridoma Bank, DSHB, Cat. #3A6B4), mouse anti-β-integrin (1:100, DSHB, Cat. #CF.6G11), mouse anti-β-Gal (1:500, DSHB, Cat. #40-1a), rabbit anti-phospho-JNK (1:200, Calbiochem, Cat. #559309), goat anti-mouse-Cy3 (1:1000, Life technologies, Cat. #A10521), and goat anti-Rabbit-Cyanine3 (1:1000, Life technologies, Cat. #A10520). Vectashield mounting media (Vector Laboratories, Cat. #H-1500) with DAPI (4,6-diamidino-2-phenylindole) was used for mounting.

### X-gal staining

Wing disks were dissected from 3^rd^-instar larvae in PBST (1×PBS, pH 7.0, 0.1% Triton X-100) and stained for β-galactosidase activity as described^65^.

### Statistics

All data were collected from at least three independent experiments. The results were presented as bar graphs created with GraphPad Prism 8.0.2. For statistical significance, one-way ANOVA with Bonferroni’s multiple-comparison test, chi-squared test or two-tailed unpaired *t*-test was applied. *P* value less than 0.05 was considered significant and center values as the mean. Error bars indicated standard deviation. ns means not significant, *P* ≥ 0.05; * is *P* < 0.05; ** is *P* < 0.01; *** is *P* < 0.001; **** is *P* < 0.0001. *P* values are included in the relevant figure legends.

## Supplementary information

Supplementary file

Supplementary F1

Supplementary F2

Supplementary F3

Supplementary F4

Supplementary F5

Supplementary F6
