# Supplementary material for: CtBP modulates Snail-mediated tumor invasion in Drosophila
Source: Cell Death Discov. 2021 Aug 4;7:202. doi: 10.1038/s41420-021-00516-x (PMC8339073; doi:10.1038/s41420-021-00516-x)
Supplement: Supplementary file 1 — Supplementary file [file 41420_2021_516_MOESM1_ESM.docx]

**CtBP modulates Snail-mediated tumor invasion in *Drosophila***

Chenxi Wu^1,2#^, Xiang Ding^1#^, Zhuojie Li^1^, Yuanyuan Huang^1^, Qian Xu^3^, Rui Zou^1^, Mingyang Zhao^1^, Hong Chang^2^, Chunhua Jiang^2^, Xiaojin La^2^, Gufa Lin^4^, Wenzhe Li^1*^ and Lei Xue^1*^

^1^ The First Rehabilitation Hospital of Shanghai, Shanghai Key Laboratory of Signaling and Diseases Research, School of Life Science and Technology, Tongji University, 1239 Siping Road, Shanghai, 200092, China.

^2^ College of Traditional Chinese Medicine, North China University of Science and Technology, 21 Bohai Road, Tangshan 063210, China

^3^ College of Integrative Medicine, Fujian University of Traditional Chinese Medicine, Fuzhou 350122, China

^4^ Key Laboratory of Spine and Spinal Cord Injury Repair and Regeneration of Ministry of Education, Orthopaedic Department of Tongji Hospital, School of Life Sciences and Technology, Tongji University

^#^ These authors contribute equally to this work

^*^ Correspondence: [lei.xue@tongji.edu.cn](mailto:lei.xue@tongji.edu.cn); [lwz@tongji.edu.cn](mailto:lwz@tongji.edu.cn)

**Supplementary Information**

Supplementary Figures

Detailed Genotypes

**Supplementary Information**

**Figure S1. Knockdown *CtBP* inhibits Ras^V12^/*lgl^-/-^*-induced extended larval stage.**

Fluorescent images showing *Drosophila* larval or pupal whole bodies (**a**-**g**). Compared with the control (**a**), Ras^V12^/*lgl^-/-^*-induced extended larval period (**c**) was strongly suppressed by expressing Puc (**e**), or depleting *CtBP* (**f**), but not expressing LacZ (**d**). Clones overexpressing Ras^V12^ (**b**) or depleting *CtBP* (**g**) did not block pupariation. Statistical analysis of the pupariation percentage (**h**) as shown in figures **a**-**g** (**a**, 100%, n=45; **b**, 100%, n=43; **c**, 0.00%, n=67; **d**, 2.56%, n=39; **e**, 96.83%, n=63; **f**, 32.69%, n=52; **g**, 100%, n=56) respectively. Chi-squared test was applied to compute *P*-values, ^***^*P*<0.001; ns, no significant difference. Scale bar: 100µm (**a**-**g**). Genotypes: (**a**) *ey*-Flp *act*>*y^+^*>GAL4 *UAS*-GFP/+, (**b**) *ey*-Flp *act*>*y^+^*>GAL4 *UAS*-GFP/*+*; *UAS*-Ras^V12^/+, (**c**) *yw ey*-Flp/+; *tub*-GAL80 FRT40A/*lgl^4^* FRT40A *UAS*-Ras^V12^; *act*>*y^+^*>GAL4 *UAS*-GFP/*+*, (**d**) *yw ey*-Flp/+; *tub*-GAL80 FRT40A/*lgl^4^* FRT40A *UAS*-Ras^V12^; *act*>*y^+^*>GAL4 *UAS*-GFP/*UAS*-LacZ, (**e**) *yw ey*-Flp/+; *tub*-GAL80 FRT40A/*lgl^4^* FRT40A *UAS*-Ras^V12^; *act*>*y^+^*> GAL4 *UAS*-GFP/*UAS*-Puc, (**f**) *yw ey*-Flp/+; *tub*-GAL80 FRT40A/*lgl^4^* FRT40A *UAS*-Ras^V12^; *act*>*y^+^*>GAL4 *UAS*-GFP/*UAS*-*CtBP*-*IR^32889^*, (**g**) *ey*-Flp *act*>*y^+^*>GAL4 *UAS*-GFP/*+*; *UAS*-*CtBP*-*IR^32289^*/+

**Figure S2. Ras^V12^/*lgl^-/-^* promotes CtBP expression and JNK activation.**

Histogram showing the levels of *CtBP* mRNA as measured by qRT-PCR (**a**-**b**). Total RNA of *Drosophila* third instar larvae eye discs were extracted and normalized for cDNA synthesis. Error bars represents standard deviation from three independent experiments. One-way ANOVA with Bonferroni multiple comparison test was used to compute *P*-values, ^**^*P*<0.01, ^***^*P*<0.001, ns, no significant difference. (**c**-**e**) Fluorescence micrographs of larval CCs stained with anti-β-gal antibody are shown. The individual channels detecting only GFP (green, **c’**-**e’**) or β-gal (red, **c’’**-**e’’**). Compared with the control (**c**) and Ras^V12^ alone (**d**), Ras^V12^/*lgl^-/-^* tumor cells exhibit strong up-regulation of *puc*-lacZ expression (**e**). (**f**-**g**) Fluorescence micrographs of clones in 3^rd^ instar larval eye disc stained with anti-cDcp-1 antibody are shown. The individual channels detecting only GFP (green, **f’** and **g’**) or cDcp-1 (red, **f’’**and **g’’**). Compared with the control (**f**), *CtBP* mutant clones (marked by GFP) did not trigger apoptosis (**g**). Scale bar: 100µm (**c**-**g**). Genotypes: (**a**) from left to right: *hs-*Gal4, *hs-*Gal4/*UAS-CtBP-IR^32889^*, *hs-*Gal4/*UAS-CtBP-IR^31334^*; (**b**) from left to right: *ey*-Flp *act>y+>GAL4 UAS*-GFP/+, *ey*-Flp *act>y+>GAL4 UAS*-GFP/+; *UAS*-Ras^V12^/+, *yw ey*-Flp/+; *tub*-GAL80 FRT40A/*lgl^4^* FRT40A *UAS*-Ras^V12^; *act*>*y^+^*>GAL4 *UAS*-GFP/*+*, (**c**) *ey*-Flp *act>y+>GAL4 UAS*-GFP/+; *puc-*LacZ/+, (**d**) *ey*-Flp *act>y+>GAL4 UAS*-GFP/+; *puc-*LacZ/*UAS-*Ras^V12^, (**e**) *yw ey*-Flp/+; *tub*-GAL80 FRT40A/*lgl^4^* FRT40A *UAS*-Ras^V12^; *act*>*y^+^*>GAL4 *UAS*-GFP/*puc*-LacZ, (**f**) *ey*-Flp *act>y+>GAL4 UAS*-GFP/+; *FRT82B tub-*Gal80/*FRT82B*, (**g**) *ey*-Flp *act>y+>GAL4 UAS*-GFP/+; *FRT82B tub-*Gal80/*FRT82B CtBP^87De-10^.*

**Figure S3. Depletion-of-*CtBP* clones show no obvious defects.**

Fluorescent images showing *Drosophila* larval CCs (**a**-**c**) and VNC (**d**-**f**), the anterior is to the up in all panels. Compared with control (**a**, **d**), loss-of-*CtBP* clones showed no obvious defects (**b**, **c**, **e** and **f**). Statistical analysis of the invasion percentage (**g**) as shown in figures **a**-**c** (**a**, 0.00%, n=52; **b**, 0.00%, n=61; **c**, 0.00%, n= 47) respectively. Scale bar: 100µm (**a**-**f**). Genotypes: (**a**, **d**) *ey*-Flp *act*>*y^+^*>GAL4 *UAS*-GFP/+, (**b**, **e**) *ey*-Flp *act*>*y^+^*>GAL4 *UAS*-GFP/+; *UAS*-*CtBP*-*IR^32889^*/+, (**c**, **f**) *ey*-Flp *act*>*y^+^*>GAL4 *UAS*-GFP/+; *UAS*-*CtBP*-*IR^31334^*/+.

**Figure S4. Loss of *CtBP* does not impair cell proliferation.**

Fluorescent micrographs of clones in 3^rd^ instar larval eye disc stained with anti-PH3 antibody are shown. Compared with the control (**a**), cell proliferation was not reduced in *CtBP* mutant clones (marked by GFP) (**b**). Scale bar: 100µm. Genotypes: (**a**) *ey*-Flp *act>y+>GAL4 UAS*-GFP/+; *FRT82B tub-*Gal80/*FRT82B*, (**b**) *ey*-Flp *act>y+>GAL4 UAS*-GFP/+; *FRT82B tub-*Gal80/*FRT82B CtBP^87De-10^.*

**Figure S5. Sna regulates loss-of-*scrib*-induced cell death.**

(**a**-**d**) Fluorescent micrographs of 3^rd^ instar larval wing discs stained with anti-cDcp-1 antibody are shown, anterior is to the left and cells are marked with GFP expression. Compared with the control (**a**-**a’’**), loss-of-*scrib* induced massive apoptotic cell death (**b**-**b’’**), which was suppressed by *sna-IR* (**c**-**c’’**). Expression of Sna also triggered apoptosis (**d**-**d’’**). Scale bar: 100µm (**a**-**d**). Genotypes: (**a**) *ptc*-GAL4 *UAS*-GFP/+, (**b**) *ptc*-GAL4 *UAS*-GFP/*UAS*-*scrib*-*IR*, (**c**) *ptc*-GAL4 *UAS*-GFP/*UAS*-*scrib*-*IR*; *UAS-sna-IR^V^*/+, (**d**) *ptc*-GAL4 *UAS*-GFP/*UAS*-Sna^74b^.

**Figure S6. Sna-induced cell invasion is independent of cell death and AiP.**

(**a**-**c**) Fluorescent micrographs of 3^rd^ instar larval wing discs stained with anti-MMP1 antibody are shown. The individual channels detecting only GFP (green, **a’**-**c’**) or MMP1 (red, **a’’**-**c’’**). Ectopic expression of Sna induced cell migration and MMP1 upregulation (**a**), which were not suppressed by expression of Dronc^DN^ (**b**) or P35 (**c**). Scale bar: 100µm (**a**-**c**). (**a**) *ptc*-GAL4 *UAS*-GFP/*UAS*-Sna^74b^; *puc*-LacZ/+; (**b**) *ptc*-GAL4 *UAS*-GFP/*UAS*-Sna^74b^; *puc*-LacZ/*UAS*-Dronc^DN^, (**c**) *ptc*-GAL4 *UAS*-GFP/*UAS*-Sna^74b^; *puc*-LacZ/*UAS*-P35.

**Genotypes in figures:**

**Figure 1**

(**a** and **i**) *ey*-Flp *act*>*y^+^*>GAL4 *UAS*-GFP/+

(**b** and **j**) *ey*-Flp *act*>*y^+^*>GAL4 *UAS*-GFP/*+*; *UAS*-Ras^V12^/+

(**c** and **k**) *yw ey*-Flp/+; *tub*-GAL80 FRT40A/*lgl^4^* FRT40A *UAS*-Ras^V12^; *act*>*y^+^*>GAL4 *UAS*-GFP/*+*

(**d** and **l**) *yw ey*-Flp/+; *tub*-GAL80 FRT40A/*lgl^4^* FRT40A *UAS*-Ras^V12^; *act*>*y^+^*>GAL4 *UAS*-GFP/*UAS*-LacZ

(**e** and **m**) *yw ey*-Flp/+; *tub*-GAL80 FRT40A/*lgl^4^* FRT40A *UAS*-Ras^V12^; *act*>*y^+^*> GAL4 *UAS*-GFP/*puc^E69^*

(**f** and **n**) *yw ey*-Flp/+; *tub*-GAL80 FRT40A/*lgl^4^* FRT40A *UAS*-Ras^V12^; *act*>*y^+^*>GAL4 *UAS*-GFP/*UAS*-Puc

(**g** and **o**) *yw ey*-Flp/+; *tub*-GAL80 FRT40A/*lgl^4^* FRT40A *UAS*-Ras^V12^; *act*>*y^+^*>GAL4 *UAS*-GFP/*UAS*-*CtBP*-*IR^32889^*

(**h** and **p**) *yw ey*-Flp/+; *tub*-GAL80 FRT40A/*lgl^4^* FRT40A *UAS*-Ras^V12^; *act*>*y^+^*>GAL4 *UAS*-GFP/*UAS*-*CtBP*-*IR^31334^*

**Figure 2**

(**a**) *ptc*-GAL4 *UAS*-GFP/+

(**b**) *ptc*-GAL4 *UAS*-GFP/*UAS*-*scrib*-*IR*

(**c**) *ptc*-GAL4 *UAS*-GFP/*UAS*-*scrib*-*IR*; *UAS-*LacZ/+

(**d**) *ptc*-GAL4 *UAS*-GFP/*UAS*-*scrib*-*IR*; *UAS-CtBP*-*IR^32889^*/+

(**e**) *ptc*-GAL4 *UAS*-GFP/*UAS*-*scrib*-*IR*; *UAS-CtBP*-*IR^31334^*/+

(**f**) *ptc*-GAL4 *UAS*-GFP/*UAS*-*scrib*-*IR*; *UAS-*Puc/+

**Figure 3**

(**a**, **c** and **e**) *yw ey*-Flp/+; *tub*-GAL80 FRT40A/*lgl^4^* FRT40A *UAS*-Ras^V12^; *act*>*y^+^*>GAL4 *UAS*-GFP/*UAS*-LacZ

(**b**,**d** and **f**) *yw ey*-Flp/+; *tub*-GAL80 FRT40A/*lgl^4^* FRT40A *UAS*-Ras^V12^; *act*>*y^+^*>GAL4 *UAS*-GFP/*UAS*-*sna*-*IR^V^*

(**i**) *ptc*-GAL4 *UAS*-GFP/*UAS*-*scrib*-*IR*; *UAS-*LacZ/+

(**j**) *ptc*-GAL4 *UAS*-GFP/*UAS*-*scrib*-*IR*; *UAS-sna*-*IR^V^*/+

**Figure 4**

(**a** and **f**) *ey*-Flp *act*>*y^+^*>GAL4 *UAS*-GFP/*+*; *UAS*-Ras^V12^/+

(**b** and **g**) *ey*-Flp *act*>*y^+^*>GAL4 *UAS*-GFP/*UAS*-Sna^74b^

(**c** and **h**) *ey*-Flp *act*>*y^+^*>GAL4 *UAS*-GFP/*UAS*-Sna^74b^; *UAS*-Ras^V12^/+

(**d** and **i**) *ey*-Flp *act*>*y^+^*>GAL4 *UAS*-GFP/*UAS*-Sna^74b^; *UAS*-Ras^V12^/*UAS-CtBP*-*IR^32889^*

(**e** and **j**) *ey*-Flp *act*>*y^+^*>GAL4 *UAS*-GFP/*UAS*-Sna^74b^; *UAS*-Ras^V12^/*UAS-CtBP*-*IR^31334^*

**Figure 5**

(**a**, **f**, **k** and **p**) *ptc*-GAL4 *UAS*-GFP/+

(**b**, **g**, **l** and **q**) *ptc*-GAL4 *UAS*-GFP/*UAS*-Sna^74b^; *UAS*-LacZ/+

(**c**, **h**, **m** and **r**) *ptc*-GAL4 *UAS*-GFP/*UAS*-Sna^74b^; *UAS-CtBP*-*IR^32889^*/+

(**d**, **i**, **n** and **s**) *ptc*-GAL4 *UAS*-GFP/*UAS*-Sna^74b^; *UAS-CtBP*-*IR^31334^*/+

(**e**, **j**, **o** and **t**) *ptc*-GAL4 *UAS*-GFP/*UAS*-CtBP^WT^

**Figure 6**

**(a)** *pnr*-GAL4/+

**(b)** *pnr*-GAL4/*UAS*-*sna*-*IR^V^*

**(c)** *pnr*-GAL4 *UAS*-*sna*-*IR^V^*/*CtBP^87De-10^*

**(d)** *pnr*-GAL4/*CtBP^87De-10^*

**(e)** *pnr*-GAL4/*UAS*-*dFoxO*-*IR*

**(f)** *pnr*-GAL4/*UAS*-*CtBP*-*IR^32289^*

**(g)** *sna^18^*/+; *pnr*-GAL4/*UAS*-*CtBP*-*IR^32289^*

**(h)** *sna^18^*/+; *pnr*-GAL4/*+*

**Figure 7**

(**a** and **d**) *ptc*-GAL4 *UAS*-GFP/+; *puc*-LacZ/+

(**b** and **e**) *ptc*-GAL4 *UAS*-GFP/*UAS*-Sna^74b^; *puc*-LacZ/+

(**c** and **f**) *ptc*-GAL4 *UAS*-GFP/*UAS*-Sna^74b^; *puc*-LacZ/*UAS*-Bsk^DN^

(**g**) *ptc*-GAL4 *UAS*-GFP *TRE*-RFP/*+*

(**h)** *ptc*-GAL4 *UAS*-GFP *TRE*-RFP/*UAS*-Sna^74b^

(**i**) *ptc*-GAL4 *UAS*-GFP/+

(**j)** *ptc*-GAL4 *UAS*-GFP/*UAS*-Sna^74b^
