# Supplementary figures and images for: CtBP modulates Snail-mediated tumor invasion in Drosophila
Source: Cell Death Discov. 2021 Aug 4;7:202. doi: 10.1038/s41420-021-00516-x (PMC8339073; doi:10.1038/s41420-021-00516-x)

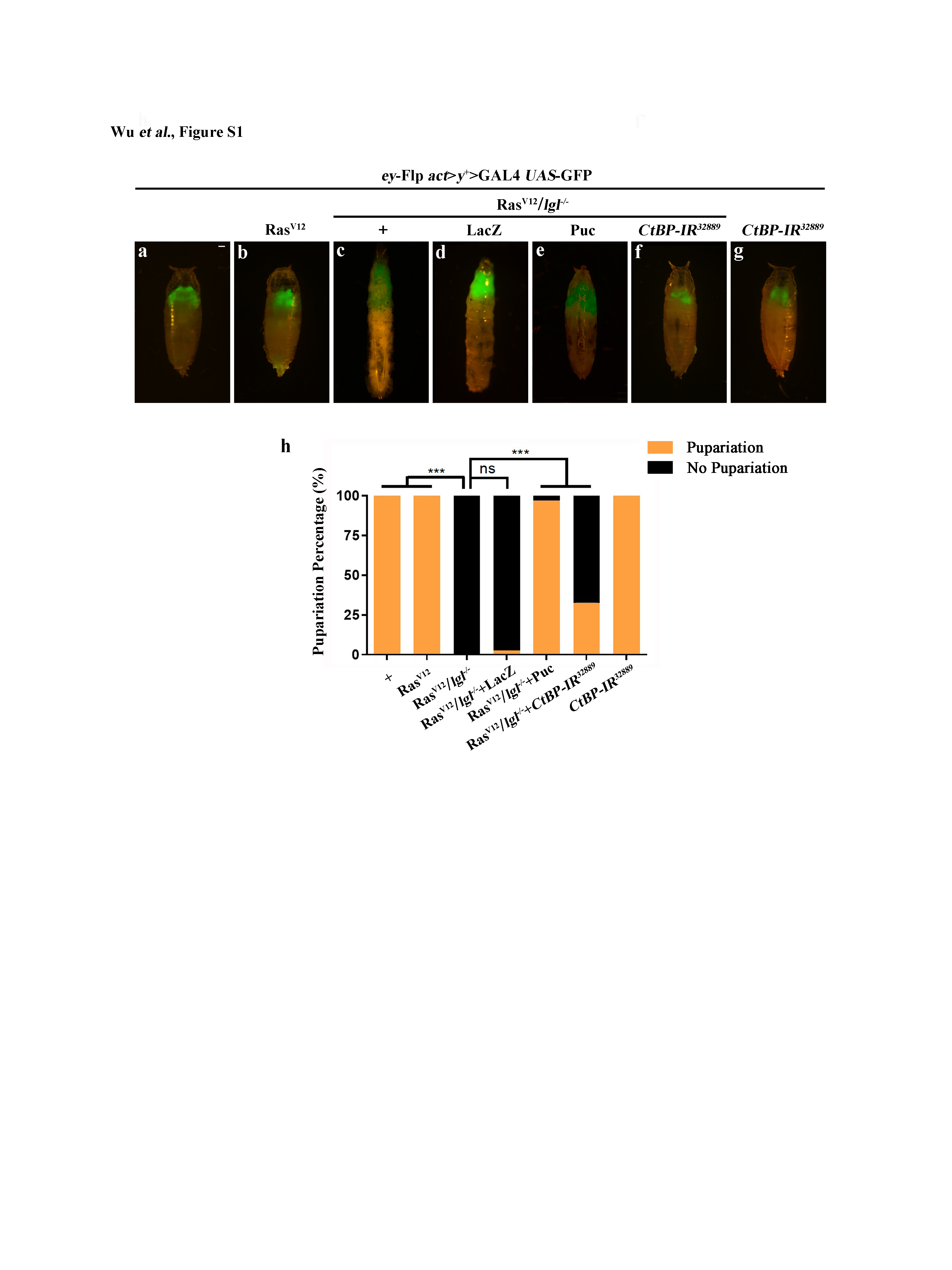

Supplement: Supplementary file 2 — Supplementary F1 [file 41420_2021_516_MOESM2_ESM.png]

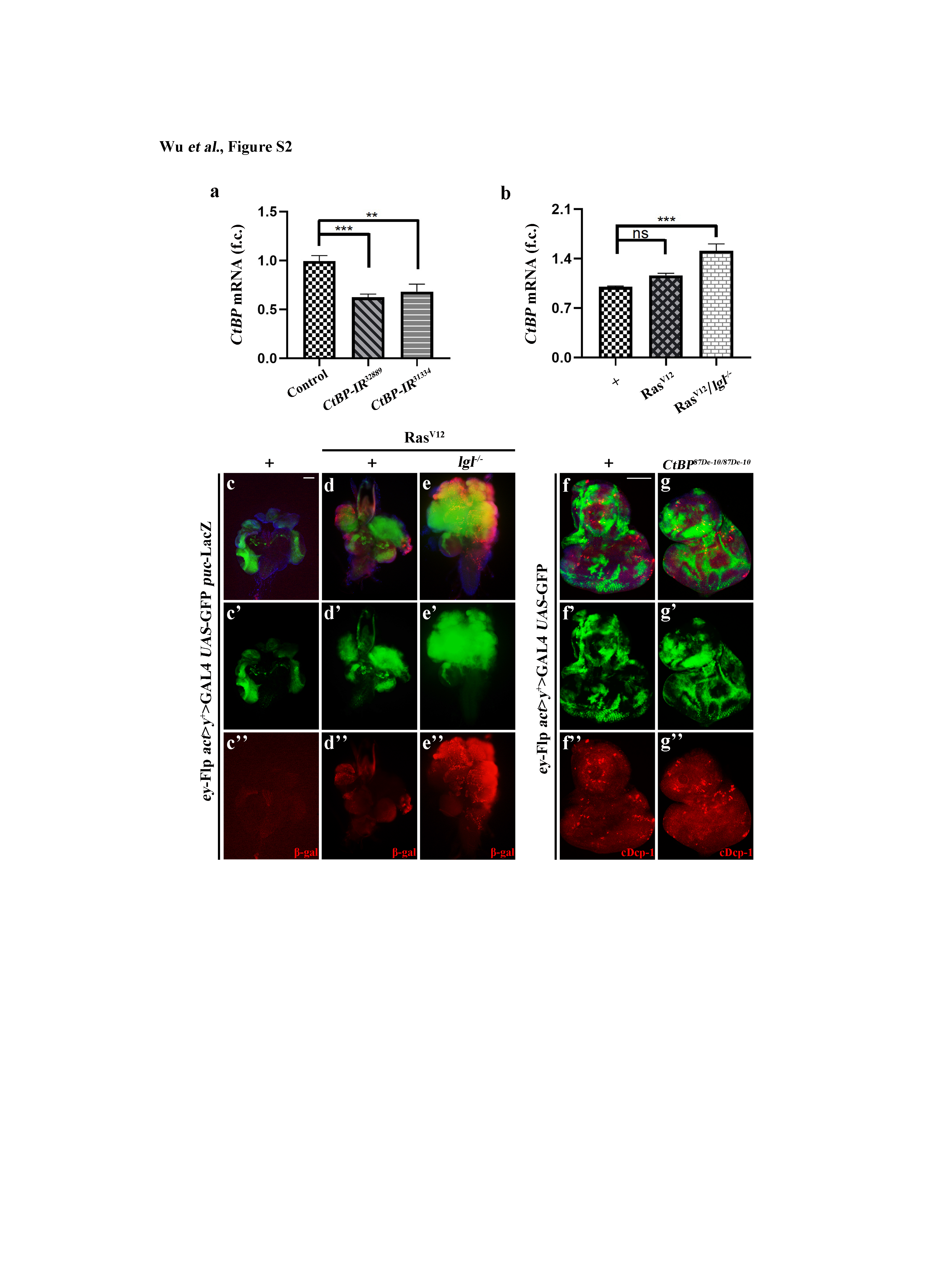

Supplement: Supplementary file 3 — Supplementary F2 [file 41420_2021_516_MOESM3_ESM.png]

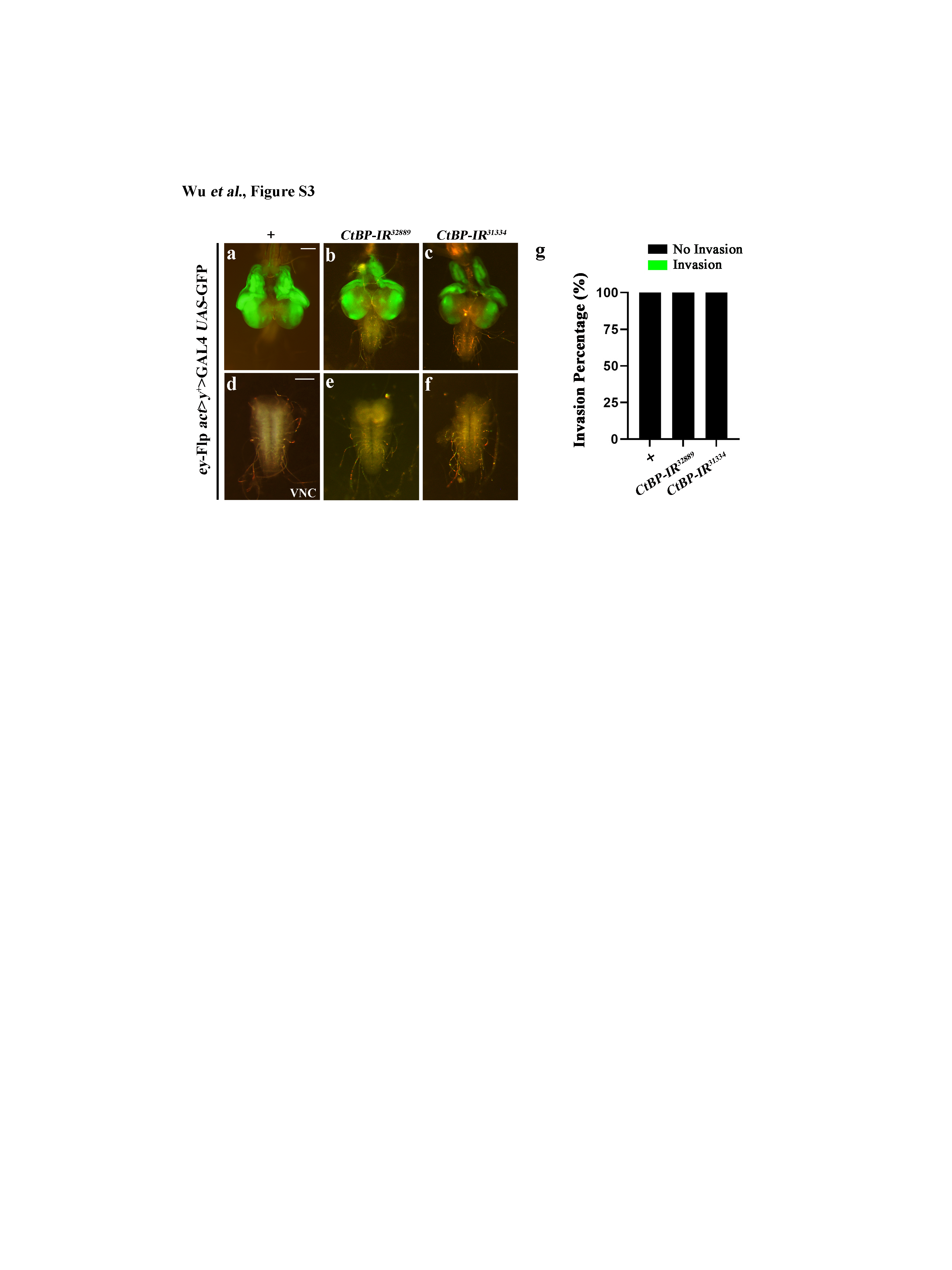

Supplement: Supplementary file 4 — Supplementary F3 [file 41420_2021_516_MOESM4_ESM.png]

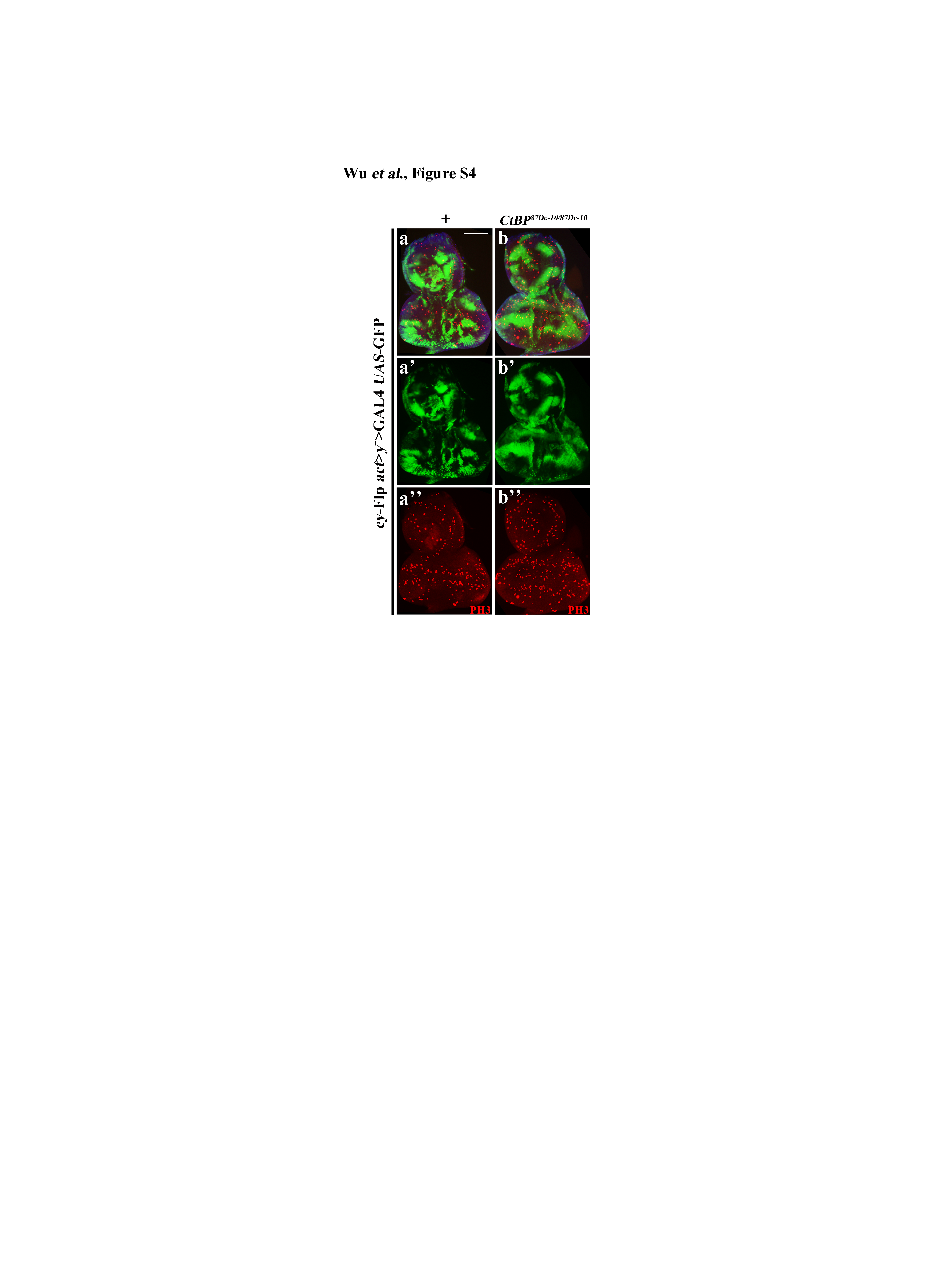

Supplement: Supplementary file 5 — Supplementary F4 [file 41420_2021_516_MOESM5_ESM.png]

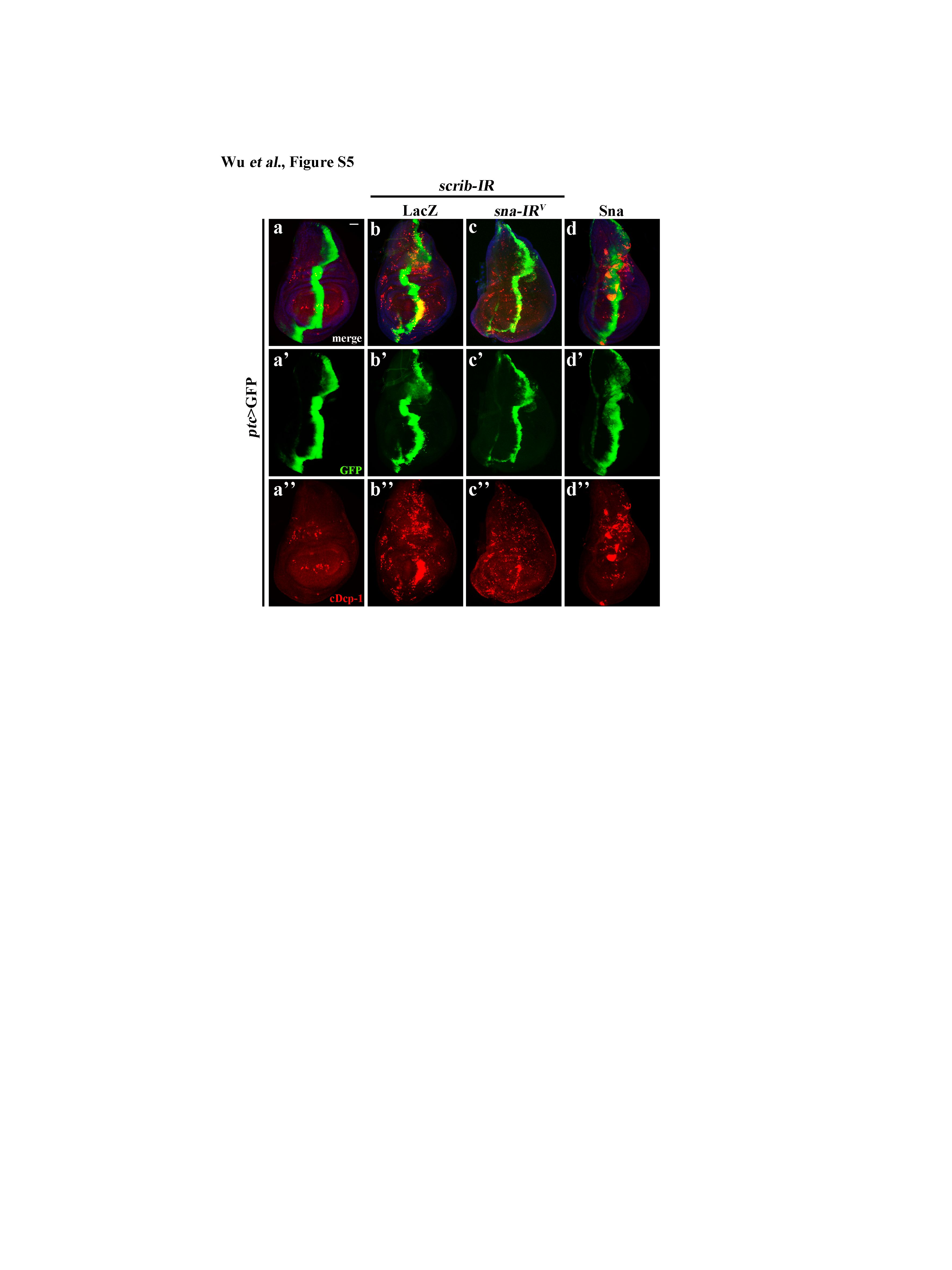

Supplement: Supplementary file 6 — Supplementary F5 [file 41420_2021_516_MOESM6_ESM.png]

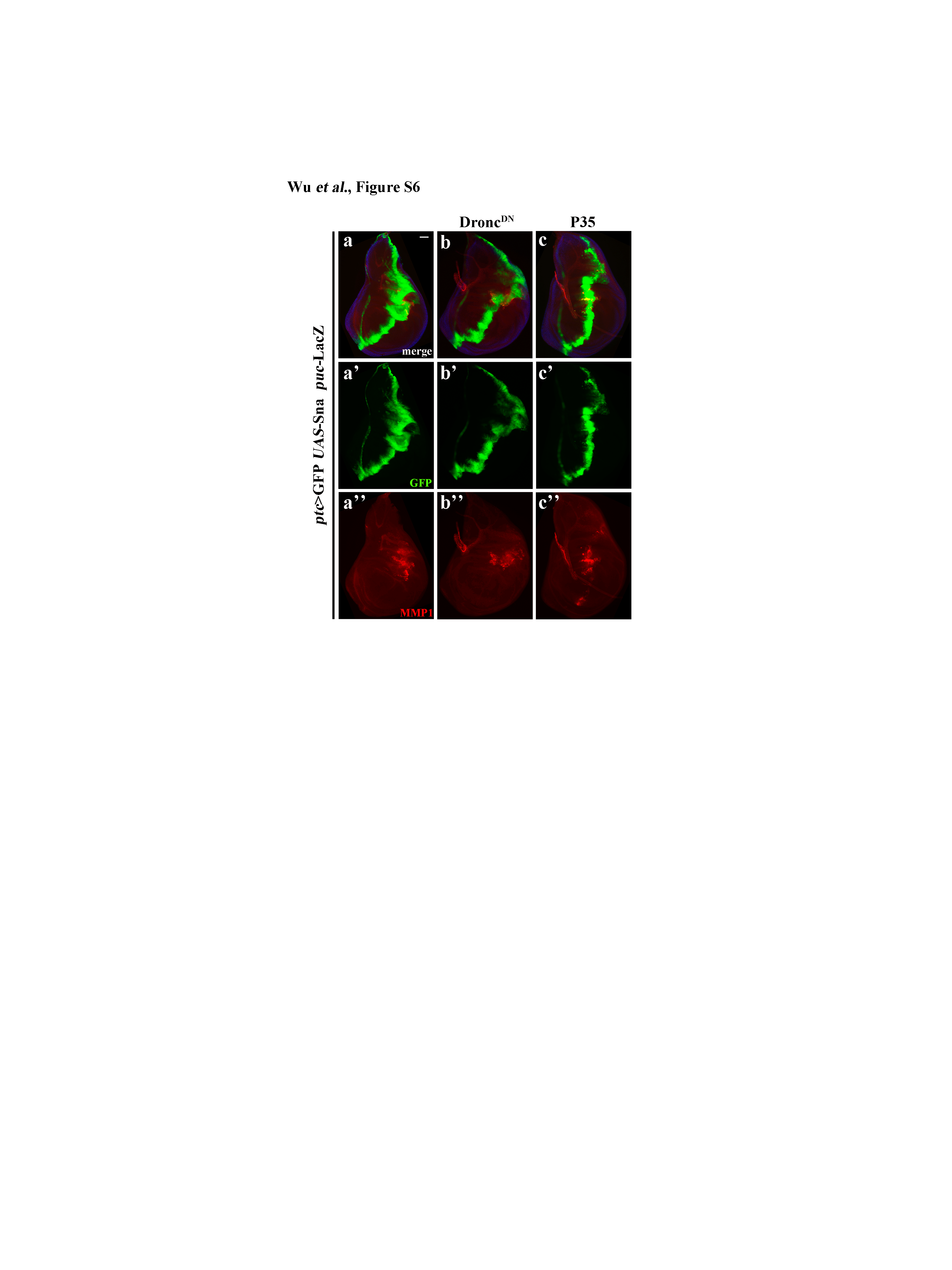

Supplement: Supplementary file 7 — Supplementary F6 [file 41420_2021_516_MOESM7_ESM.png]
